# Unlimited Thirst for Genome Sequencing, Data Interpretation, and Database Usage in Genomic Era: The Road towards Fast-Track Crop Plant Improvement

**DOI:** 10.1155/2015/684321

**Published:** 2015-03-19

**Authors:** Arun Prabhu Dhanapal, Mahalingam Govindaraj

**Affiliations:** ^1^Division of Plant Sciences, University of Missouri, Columbia, MO 65211, USA; ^2^International Crops Research Institute for the Semi-Arid Tropics (ICRISAT), Patancheru 502324, India

## Abstract

The number of sequenced crop genomes and associated genomic resources is growing rapidly with the advent of inexpensive next generation sequencing methods. Databases have become an integral part of all aspects of science research, including basic and applied plant and animal sciences. The importance of databases keeps increasing as the volume of datasets from direct and indirect genomics, as well as other omics approaches, keeps expanding in recent years. The databases and associated web portals provide at a minimum a uniform set of tools and automated analysis across a wide range of crop plant genomes. This paper reviews some basic terms and considerations in dealing with crop plant databases utilization in advancing genomic era. The utilization of databases for variation analysis with other comparative genomics tools, and data interpretation platforms are well described. The major focus of this review is to provide knowledge on platforms and databases for genome-based investigations of agriculturally important crop plants. The utilization of these databases in applied crop improvement program is still being achieved widely; otherwise, the end for sequencing is not far away.

## 1. Introduction

Most recent development of high-throughput methods for analyzing the structure and function of genes is collectively referred to as “genomics.” The comprehensive information of this kind is currently available for only a few plants and is rapidly being available for most of the higher plants and several underutilized crop plant species. Public access to this information will exploit biological selections and have direct impact on application of genomics to the improvement of economically important plants. Getting sequences of major plants on the one hand and access to all sequenced information for further applications on the other hand are most important. Therefore, global biological community should have open-access database for all plant genome sequenced so far.

Plant databases are facilities or long-lived record that are systematically updated with massive amount of data which has been generated as research outcomes in the context of the whole field of plant biology to ensure maximal accessibility and visibility to use by researchers in different fields of interest. These databases assist in drawing conclusion to make some new hypotheses to address basic questions of researchers. Internet-accessible information has become an integral part of most scientific enterprise, including the plant sciences. It now seems that it is impossible to conceive of future significant progress being made without the internet and the databases and many other similar resources the internet makes openly available. This is particularly true as the information flows from genomics and other high-throughput technologies to all aspects of crop plant sciences. The ultimate goal of plant genomics is to improve our ability to identify the genotypes with optimal agronomic traits in order to improve yield, a must with the increasing world population [1].

## 2. Omics Research on Crop Plants: Present Status

**Figure 1:**
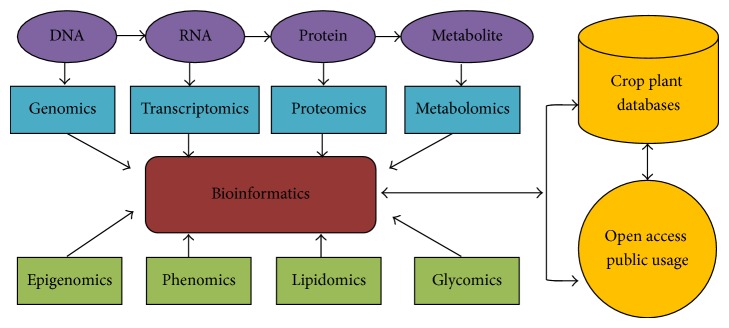
Flowchart description to integration of biological omics platforms with bioinformatics linking crop plant databases and open-access public usage.

“Omics” refers to the collective technologies that are made available in recent years which are used to explore the roles, relationships, and actions of the various types of molecules that make up the cells of a living organism. The “omics” technology includes genomics (the study of genes and their function), proteomics (the study of proteins), metabolomics (the study of molecules involved in cellular metabolism), transcriptomics (the study of the mRNA), glycomics (the study of cellular carbohydrates), and lipomics (the study of cellular lipids). These omics technologies provide the tools needed to look at the differences in DNA, RNA, proteins, and other cellular molecules between species and among individuals of the same or different species. A combinatorial approach using multiple omics platforms and integration of their outcomes is now an effective strategy for clarifying molecular systems integral to improving crop plant productivity (Figure 1). Recent progress in plant genomics and utilization of genetic resources has allowed us to discover and isolate important genes and analyze their functions that regulate yields as well as stress tolerance [2].

A technological advance in omics research integrating animal and plant science has become essential resources for the investigation of gene function in association with phenotypic changes. Some of these advances include the development of high-throughput methods for profiling expressions of thousands of genes, for identifying modification events and interactions in the plant proteome and for measuring the abundance of many metabolites simultaneously. In addition, large-scale collections of bioresources, such as mass-produced mutant lines and clones of full-length cDNAs and their integrative relevant databases, are now made available [3, 4]. The importance of crop plant genetic resources and insights that have been emerged in recent years through genomics are well reviewed [5, 6]. The recent high-throughput technological advances have provided opportunities to develop collections of sequence-based resources and other related resource platforms for specific organisms. Various bioinformatics platforms have become essential tools for accessing omics dataset for the efficient mining and integration of biologically significant knowledge to deposit in databases for public access (Figure 1).

## 3. Crop Plant Genome Sequence Resources

In recent years, many crop plant genomes have been sequenced and data is available to public (Table 1). On the other hand, collected sequence data provide essential genomic resources for accelerating molecular understanding of biological properties and for promoting the application of such knowledge to the benefit of humans. The recent accumulation of nucleotide sequences of model plants and other crop species has provided fundamental information for the design of sequence-based research applications in functional genomics. Species-specific nucleotide sequence collections also provide opportunities to identify the genomic aspects of phenotypic characters based on genome-wide comparative analyses and knowledge of model organisms [46].

### 3.1. Rationale of Genome Sequencing Projects

Recent revolution in DNA sequencing technology has brought down the cost of DNA sequencing of several crop plant species and made the sequencing of an increased number of genomes both feasible and cost effective [46]. The first plant genome* Arabidopsis* was completely sequenced in December 2000, and it was the third complete genome of a higher eukaryote and further studies were carried out in recent years on* Arabidopsis thaliana* and* Arabidopsis lyrata* [30, 31]. Subsequently, after Arabidopsis, several other crop plants have been sequenced (Table 1). These genomes reveal numerous species-specific details, including genome size, gene number, patterns of sequence duplication, a catalog of transposable elements, and syntenic relationships. To understand the complex instructions contained in all these raw sequence information of the plant genome, large-scale functional genomics projects are required. Progress towards a complete understanding of gene regulatory networks shared among many crop plants is important for improving cultivated species and for complete understanding of crop plant evolution.

### 3.2. Contribution of Whole-Genome Resequencing

Advancement in next-generation sequencing (NGS) technology coupled with many reference genomes sequence data allows us to discover variations among many crop plants. A whole-genome resequencing project to discover whole-genome sequence variations in 1,001 strains (accessions) of* Arabidopsis* resulted in dataset that became a fundamental resource for promoting future genetics studies to identify alleles in association with phenotypic diversity across the entire genome and across the entire crop plant species (http://1001genomes.org/) [47, 48]. In rice, a high-throughput method for genotyping recombinant populations that used whole-genome resequencing data generated by the Illumina Genome Analyzer was performed [18] and recently resequencing of 50 accessions of cultivated and wild rice yields markers for identifying agronomically important genes has been completed [49].

### 3.3. Analyzing Crop Plant Genome Sequences

Galaxy (http://galaxyproject.org) is a software system that provides knowledge and support through a framework that provides researchers with simple interfaces to powerful data interpretation tools. Galaxy is web-based framework designed for use of experimental and computational biologists in all fields of biological science. With Galaxy, one can easily use analysis tools through a web-based interface [50]. Another tool made available from the Sanger institute (http://www.sanger.ac.uk/) is Artemis, a free genome browser and annotation tool that allows visualization of sequence features, next generation data, and the results of analyses [51]. The Broad's Genome Sequencing and Analysis Program (GSAP) plays a major role in providing several analyses tools for genome sequences coming out of the NGS platforms in all biological fields (http://www.broadinstitute.org/).

## 4. Crop Plant Genome Resources and Variation Analysis

Genome-wide study of both structural and gene content variation are hypothesized to drive important phenotypic variation within a crop plant species. Previous studies have shown that both structural and gene content variations were assessed in several crops using array hybridization and targeted resequencing. Genetic variation within and between species is most commonly quantified by single nucleotide polymorphisms (SNPs). There has been increased interest in recent years to resolve genetic differences in terms of structural variation (SV), which includes copy number variation (CNV) caused by large insertions and deletions, and other types of rearrangements such as inversions and translocations. CNV together with SV is thought to be an important factor in determining phenotypic variation for a wide range of traits reviewed [52] in both crop plant and animal species.

### 4.1. Molecular Breeding Tools

#### 4.1.1. Role of Molecular Markers

Among various DNA markers available to research community, single sequence repeats (SSRs) and single nucleotide polymorphisms (SNPs) are most widely used today. SSRs are demonstrated to be of high degree of transferability between species and could easily be transferred to related species to amplify the same corresponding locus. SNPs represent the most frequent type of genetic polymorphism and may therefore provide a high density of markers near a locus of interest compared to SSRs. The high density of SNPs makes them valuable for genome mapping, and in particular they allow the generation of ultra-high density genetic maps and haplotyping systems for genes or regions of interest and map-based positional cloning in crop plants. SNPs are used routinely in crop breeding programs, for genetic diversity analysis, cultivar identification, phylogenetic analysis, characterization of genetic resources, and association with agronomic and physiological traits in both cereals and legumes [53, 54]. Application of SNP markers for genetic dissection of complex traits like delta ^13^C and delta ^15^N in legume like soybean with high density SNP chips has also increased and been made available [55–57].

#### 4.1.2. Biparental QTL Mapping

The quantitative traits loci (QTL) identified for a trait of interest that contribute to higher phenotypic variation are considered major QTL. These identified QTLs, after validation in desired germplasm, can be used for introgression of the trait from the donor genotypes (generally used for identification of the QTL for the trait) into elite cultivars to traits of less phenotypic variation cultivars or breeding lines (recipient parents) without transfer of undesirable genes from the donors (linkage drag). The process is commonly referred to as marker-assisted backcrossing (MABC) most commonly employed by plant breeders. Superior lines or cultivars are developed which contain only the major QTL from the donor parent while retaining the whole-genome of the recurrent parent [58]. MABC has been used extensively for introgression of resistance to biotic stresses and abiotic stress in crop plants. To overcome the limitations of MABC, particularly when multiple QTLs control the expression of a complex trait, the MARS approach, which involves intermating selected individuals in each selection cycle, has been recommended [59, 60]. It generally involves the use of an F2 base population and can be used in self-pollinated crops like wheat, barley, and chickpea for developing pure lines with superior per se performance (for more details, see [60]). MARS has the additional advantage of overcoming the limitation of inadequate improvement in the frequency of superior alleles in F2 enrichment since MAS is practiced in each cycle following intermating to improve the frequency of favourable alleles [59].

#### 4.1.3. Genome-Wide Association Analysis

Genome-wide association analysis (GWAS) is a powerful approach to identify the causal genetic polymorphisms underlying both simple and complex traits in crop plants. Advancement in genomics has provided alternative tools to improve breeding efficiency in plant breeding programs. Molecular markers linked to the causal genes and/or QTLs can be used for marker-assisted selection (MAS) [61]. Recent advances in genome sequencing and single nucleotide polymorphism (SNP) genotyping have increased the applicability of association analysis for QTL mapping in crop plants [62, 63]. Genome-wide association analyses with SNP markers have been conducted for several important traits in many plant species, including* Arabidopsis thaliana* [64], maize [65], rice [66], and soybean [67–69], and also in tree crops like peach [70].

#### 4.1.4. Genomic Selection

Genomic selection (GS) is more reliable and relatively simple and most powerful approaches used in crop plant species where breeding values of the genotype/cultivar lines are predicted using their marker genotypes and phenotypes [71]. GS captures the small QTL effect that governs the variation including epistatic interaction effects. GS has been successfully used in wheat, maize, and soybean [71–73]. The accuracy of GS depends on genetic × environmental (G × E) interaction and major challenge of GS is to arrive with the accurate genomic estimated breeding values (GEBVs) with respect to the G × E interaction. Application of GS has been extended to other crops plants like* Arabidopsis*, sugarcane, and sugar beet in recent years.

### 4.2. Application of Molecular Platforms for Variation Analysis

High-throughput polymorphism analysis is an essential tool for facilitating any genetic map-based approach, and the number of platforms has been developed and applied to genetic map construction, marker-assisted selection, and QTL cloning using multiple segregation populations in major crop plants. These types of genotyping systems have been successfully used in postgenome sequencing era with extending of their projects on genotyping of genetic resources, identifying their population structure, and association of their phenotypic values to identify their genomic regions. This recent expansion of analysis platforms provides an essential resource in the “variome” study of crop plants. The increasing demand for high-throughput and cost-effective platforms for comprehensive variation analysis (also called variome analysis) has rapidly increased. Whole-genome resequencing approaches are already being realized as a direct solution for variome analysis in species whose reference genome sequence data are available [74, 75].

Diversity Array Technology (DArT) is a high-throughput genotyping system developed based on a microarray platform (http://www.diversityarrays.com/index.html) [76]. In various crop species such as wheat, barley, and sorghum, DArT markers have been used together with conventional molecular markers to construct denser genetic maps and perform association studies [77–79]. The Illumina GoldenGate assay allows the simultaneous analysis of up to 1,536 SNPs in 96 samples and has been used to analyze genotypes of segregation populations in order to construct genetic maps allocating SNP markers in crops such as barley, wheat, soybean [80–82], and peach [70, 83]. Recently 3K to 700K Infinium i Select HD and HTS custom genotyping bead chips are made available for the high-throughput genotyping of SNPs, indels, and CNVs.

### 4.3. Databases for Variation Analysis

Characterizing the genetic basis of variation in crop plants and linking to observable traits will provide an important framework for understanding evolutionary patterns and population structure and could specially increase the efficiency of selection made in the crop plant breeding programmes.

*GRAMENE*. The Genetic Diversity Database in GRAMENE specializes in storage of genotypes, phenotypes and their environments, germplasm, and association data. Genomic Diversity and Phenotype Data Model (GDPDM) database schema which efficiently stores anything from small-scale SSR diversity studies to large-scale SNP/indel-based genotype-phenotype studies with billions of allele calls [84].

*The Plant Variation Mart Database*. It holds a catalogue of DNA variants for single nucleotide polymorphisms (SNPs) and insertions/deletions (indels) for* Arabidopsis*, rice, and grapes.

## 5. Crop Plant Comparative Genomics Resources

The number of sequenced crop plant genomes and their associated genomic resources is growing rapidly with the advent of increased focus on crop plant genomics from funding agencies and other NGS technologies. Among several comparative genomics platform available today, Phytozome, a comparative hub to plant genome and gene family data and analysis, provides a view of the evolutionary history of every plant gene at the level of sequence, gene structure, gene family, and genome organization. Through their comprehensive plant genome database and web portal, these data are available to the broader plant science research community, providing powerful comparative genomics tools that help link model systems with other plants of economic and ecological importance. A number of information resources to plant genomics accessible on the web have appeared, along with appropriate analytical tools. The integrative databases promoting plant comparative genomics and URLs of each integrative database in plant genomics are shown (Table 2).

### 5.1. Crop Plant Comparative Genomics Databases

Several plant traits, namely, anatomical, morphological, biochemical, and physiological features of individuals or their component organs or tissues, serve as the key to understanding and predicting the adaptation of ecosystems in the face of biodiversity loss and global change. The reduced genome sequencing cost is opening up significant opportunities for crop improvement through plant breeding and increased understanding of plant biology. Many crop plant genomes are large and have complex evolutionary histories, making their analysis theoretically challenging and highly demanding of computational resources. Issues also include genome size, polyploidy, and the quantity, diversity, and dispersed nature of data in need of integration.

*Plant Trait Database*. The main focus of TRY (https://www.try-db.org/TryWeb/Home.php) database is to bring together the different plant trait databases worldwide into a comprehensive web-archive of the functional biodiversity of plants at the global scale by assembling, harmonizing, and distributing published and unpublished data on functional plant traits as well as a wide range of ancillary methodological and environmental information. It contains 3 million trait records for 750 traits of 1 million individual plants, representing 69,000 plant species [85, 86].

*TransPLANT*. Recently 11 European partners gathered to address growing database challenges and to develop a transnational database called “transplant” (http://www.transplantdb.eu/about) to help increasing database needs. Bringing together groups with strengths in data analysis, plant science, and computer science and from the academic and commercial sectors, transPLANT has developed integrated standards and services and undertaken new research and development needed to capitalize on the sequencing revolution, across the spectrum of agricultural and model plant species.

*PlantsDB*. This is another most commonly used database by various degree of researchers, and it comprises database instances for tomato,* Medicago*,* Arabidopsis*,* Brachypodium*,* Sorghum*, maize, rice, barley, and wheat. Building up on that, state-of-the-art comparative genomics tools such as CrowsNest are integrated to visualize and investigate syntenic relationships between monocot genomes. Results from novel genome analysis strategies targeting the complex and repetitive genomes of Triticeae species (wheat and barley) were provided and cross-linked with model species [87, 88].

### 5.2. Application of Comparative Genomics Platforms

Advancing genomic tools have provided higher boost for researchers in plant science community to understand the functional roles of genes and their evolutionary histories. Recently, resequencing additional genomes of a reference species has been made available [89], improving the understanding of genomic variation. Comparison of genomes gives insights into the evolution and adaptation of species to specific environments when compared to the information of genes provided by a single genome. To do comparative genomics studies there is a need of additional cost and as the number of available genomes increases, large-scale analyses become increasingly difficult for nonexperts, where need for computational biologist becomes essential [17]. Furthermore, biological variation between species and differences in sequence quality enhance the complexity of evolutionary analyses. Therefore, platforms for comparative genomics that take care of some of these challenges are valuable resources for experimental biologists [90, 91]. Comparative genomics has proven to be a valuable approach to understanding biology, not only for dissecting patterns and processes of genome evolution but also in revealing aspects of different gene function. The rapid advancement in comparative genomics technology, both for sequencing and for determining expression and interaction patterns, will continue to propel comparative genomics area of research in near future.

### 5.3. Emerging Databases for Comparative Genomics Analysis

To cope up and interact with increased data due to higher number of plant genome sequencing and inexpensive NGS technologies, recently developed and improved Phytozome database (http://www.phytozome.net) has provided a comparative hub for crop plant genome and gene family data analysis. The number of sequencing crop plant genomes is rapidly increasing and, at the same time, comparative sequence analysis has significantly changed our vision on the complexity of gene function, genome organization, and regulatory pathways. To explore all this genome information, a centralized infrastructure is required where all data generated by different sequencing initiatives is integrated and combined with advanced methods for data mining.

*PLAZA.* It is an online platform of plant comparative genomics (http://bioinformatics.psb.ugent.be/plaza/) that integrates functional and structural annotation of published crop plant genomes together with a large set of interactive tools to study gene and genome evolution along with their gene function. Precomputed datasets cover, intraspecies dot plots, whole-genome multiple sequence alignments, homologous gene families, phylogenetic trees, and genomic colinearity between species are provided by PLAZA. In conclusion, PLAZA provides the most comprehensible and up-to-date research environment to aid researchers in the exploration of genome information [92].

*GreenPhylDB*. GreenPhylDB is a component of the South Green Bioinformatics Platform (http://southgreen.cirad.fr/) and is open to public access (http://greenphyl.cirad.fr). GreenPhylDB is a database designed for functional and comparative genomics-based study on complete genomes. GreenPhylDB contains sixteen full genomes of members of the plantae kingdom, ranging from algae to angiosperms, automatically clustered into gene families. The database offers various lists of gene families including plant, phylum, and species specific gene families. Gene families are manually annotated and then analyzed phylogenetically in order to elucidate orthologous and paralogous relationships. It enables comparative genomics in a broad taxonomy context to enhance the understanding of evolutionary processes and thus tends to speed up gene discovery [91].

*iPlant Collaborative.* It enables transformative research through the use of a unified cyberinfrastructure funded by National Science Foundation (NSF) Plant Science Cyberinfrastructure Collaborative (PSCIC). iPlant (http://www.iplantcollaborative.org/) is a community of educators, researchers, and students working to enrich all plant sciences through the development of cyberinfrastructure, the physical computing resources, virtual machine resources, collaborative environment and interoperable analysis software and data services that are essential components of modern biology.

*KBase.* It (http://kbase.us/) provides an open, extensible framework for secure sharing of data, tools, and scientific conclusions in predictive and systems biology. The Department of Energy Systems Biology Knowledgebase (KBase) is an emerging software and data environment designed to enable researchers to collaboratively generate, test, and share new hypotheses about gene and protein functions and also to perform large-scale analyses on a scalable computing infrastructure and model interactions in microbes, plants, and their communities.

## 6. Cross-Talk between Different Databases

Although several databases are available to public, still there is a lack of information needed for researchers exactly for what they are looking for. The update should not only take place in individual plant databases but also in all comparative genomic databases holding the genome. Updating the new version of genome for crop plant species should be uniform with several databases holding the genomes. The crop/plant specific databases should be updated periodically with new variety/germplasm lines whenever it becomes available including the ploidy level of the genome information for the easy access to researchers. Integration of data types and sources will continue to be a struggle in the future. In addition to the technical problems with integration, there is a need for vision at all community levels as to the role of integrating databases in the crop plant sciences for better usage. Several species focused databases like Graingenes (http://wheat.pw.usda.gov/) for* triticea*, oats, and sugarcane;* Brachypodium* database (http://www.brachypodium.org/) for* B. distachyon*; MaizeGDB (http://www.maizegdb.org/) for maize; Oryzabase (http://www.shigen.nig.ac.jp/rice/oryzabase/) for rice; BRAD (http://brassicadb.org/brad/) for* Brassica* crops; Legume information system (http://www.comparative-legumes.org/) for legumes; and SOL Genomics Network (SGN) (http://solgenomics.net/) for* Solanaceae* crop species should come forward for an integrated platform for researchers in field of crop plant science. The integrated breeding platform (IBP) of iPlant collaborative (http://www.integratedbreeding.net/) is playing big role to help plant breeders accelerate the creation and delivery of new crop varieties in the context of an increasing global demand for food.

## 7. Tools Needed for Data Interpretation and Utilization for Crop Improvement

All crop plant databases should be updated with basic statistical to advanced sequence analysis tools. As the sequence information has been made available to public for several crop plant genomes. Data interpretation tools should be developed within the databases for easy access of researchers. Reality is that many potential users will not use available resources for a number of reasons including lack of basic training in the use of bioinformatics, resources too difficult to learn and extract data, and simple inertia at learning new tools. Training of scientists for the current and future bioinformatics landscape is essentially important. Part of the solution is time since younger researchers are more attuned to the importance of bioinformatics than many established researchers. But more formal training in all aspects of bioinformatics tools, including database essentials and use, should be done for all future biological scientists. Having inbuilt tools for QTL linkage mapping, association mapping, genomic selection, and many more tools will aid the plant researchers to use the tool of interest and speed up the process of crop improvement.

## 8. Need for More Applied Research in Crop Plants

Alike quantitative trait loci (QTL), the genome sequencing project has provided much of the raw data for most of model as well as cultivated crops, which has shaped our view on genetics insights and evolution over the past two decades. Since it is a well teaching stuff to understand the complete architecture of organism, however, no applied researches have been undertaken so far in many of the sequenced crops that are already available to public (i.e., research impact is as same as the presequencing era) and now such work is just pleasure to read with beautiful chromosome maps and dizzying Venn diagrams. For instance, cereal genome sequencing (rice, wheat, sorghum, etc.,) was completed, but yet no demonstrated work on the cultivar development had been published or undertaken for wider applied research. Genome papers have been the bread and butter of evolutionary biologists and geneticists for decades [93]. Everyone is jumping from one genome sequence to the next and looking to score a major publication aiming long-run project funding as some donors encourage them [93]. Everyone would like to see the genome sequencing projects in an optimistic way (any innovation takes its own time to influence the community) that can help us break some of the genetic bottle-neck for crop improvement in the early phase of 21st century. One and all, we should agree that every genome sequence project should have been deliberately designed to study the function of the gene in addition to the structural architecture for applied research since applied research is badly required for ongoing multi-sector crisis including agricultural food production under marginal lands. Product oriented research will have more impact than basic research alone. For instance, if more applied research is not undertaken then “genome-based research” could soon be dead which would affect the applied breeding for new cultivar development with respect to food crops as food security has still been a critical challenge for coming decades; populations blowing up unexpectedly in most of the developing countries and the novel agricultural research system should be in place to feed more than 9 billion people around the world in 2050.

## 9. Major Limitation of the Databases

As new sequencing technologies come online and the costs continue their downward trend, there will always be “more” worthy sequencing projects. Already we see multiple sequencing from the same genera with both the* Oryza japonica* and* Oryza indica* genomes sequenced and additional* Arabidopsis* genome projects following that of* Arabidopsis thaliana*. Making the crop plant databases and related bioinformatics tools easily accessible to research community is going to be a continual problem. As the volume of data power of computers increases, what is not possible is the software to fully use the potentials and the expertise of users in accessing those potentials. The amount of sequence data generated in crop plant research has dramatically increased over the last few years and will continue to accelerate in near future.

Researchers would want the complete genome sequence of every line of every organism under study; thus, an effectively unlimited thirst for sequence information will happen in near future. There will be whole-genomes of additional plants, the already mentioned sequence of additional versions of plant genomes, and intense resequencing of specific regions over tens, hundreds, and thousands of genomes. Custom microarrays are already made to resequence hundreds of thousands of dispersed DNA sequences. Resequencing to discover SNPs allows rapid genotyping through various array technologies. Currently, the planning is based more towards a minimal number necessary for a given program, but as cost declines and higher resolutions are within range of breeding programs, the density of desired SNPs may approach the entire genome level. There will also be more integration of data as knowledge, database, and analysis tools interlink. Functional genomics data on mRNA transcription and expression will tie to proteomic analyses and metabolomics of entire plants.

## 10. Conclusions

The implications of genomics on crop production can be envisioned on many fronts since fundamental advances in genomics would greatly accelerate the acquisition of knowledge and in turn will directly impact many aspects of the processes associated with crop plant trait improvement thereby considering productivity in a given environment. However, the complexity of possible higher orders of interactions can only be speculated with much more information, but the reasonable assumption is that it will dwarf our current limited views. A consequence of more voluminous and complex data is essential for better visualization and final validations. Better graphic tools to consolidate and summarize, and integration of data in a flexible manners to customize each researchers requirement. There will be more adoption of simultaneous data presentations and near future will involve ever more powerful computers, computational capability, sophisticated displays and interpretation tools, and greater practical expertise in the capabilities and exploitation of databases. Unless all these datasets are utilized in applied/product-oriented breeding program, the sequence data's just to stay with its obituary notes in database network. Hence, scientist needs critical attention and discussion within and among disciplinary on the applied platforms of outcomes for better recognition of their novel research for betterment of humankind.

## Figures and Tables

**Table 1:** List of crop plant genomes available to public with URL information and references.

Name of crop plant	Consortium/initiative	URL	References
Alfalfa (*Medicago sativa*)	Consortium	http://www.alfalfa-genome.org/www/	Young et al., 2011 [7]

Apple (*Malus domestica*)	Consortium	http://www.rosaceae.org/species/malus/malus_spp	Velasco et al., 2010 [8]

Banana (*Musa acuminate*)	The Global Musa Genomics Consortium	http://www.musagenomics.org/	D'Hont et al., [9]

Barley (*Hordeum vulgare*)	International Barley Genome Sequencing Consortium	http://www.public.iastate.edu/~imagefpc/IBSC%20Webpage/IBSC%20Template-home.html	The International Barley Genome Sequencing Consortium [10]

Cacao (*Theobroma cacao*)	Consortium	http://www.cacaogenomedb.org/	Argout et al., 2011 [11]

Cannabis (*Cannabis sativa*)	Consortium	http://genome.ccbr.utoronto.ca/index.html?org=C.+sativa&db=canSat3&hgsid=11252	van Bakel et al., 2011 [12]

Castor bean (*Ricinus communis*)	TIGR	http://castorbean.jcvi.org/	Chan et al., 2010 [13]

Chickpea (*Cicer arietinum*)	Consortium (ICRISAT-BGI)	http://www.icrisat.org/gt-bt/ICGGC/GenomeManuscript.htm	Varshney et al., 2013 [14]

Chocolate (*Theobroma cacao) *	Consortium	http://www.cacaogenomedb.org/main	Argout et al., 2011 [11]

Cotton (Gossypium * raimondii*)	BGI	http://www.cottondb.org/wwwroot/cdbhome.php	Wang et al., 2012 [15]

Common bean (*Phaseolus vulgaris* L.)	Consortium	http://www.phytozome.net/commonbean.php	Schmutz et al., 2014 [16]

Crucifer (*Thellungiella parvula*)	Consortium	http://www.brassica.info/info/events.php	Dassanayake et al., 2011 [17]

Cucumber (*Cucumis sativus*)	International Cucurbit Genomics Initiative (ICuGI)	http://www.icugi.org/	Huang et al., 2009 [18]

Date palm (*Phoenix dactylifera*)	Consortium	http://qatar-weill.cornell.edu/research/datepalmGenome/download.html	Al-Dous et al., 2011 [19]

Wheat (*Triticum aestivum*)	International Wheat Genome Sequencing Consortium (IWGSC)	http://www.wheatgenome.org/	International Wheat Genome Sequencing Consortium, 2014 [20]

Foxtail millet (*Setaria italica*)	Beijing Genomics Institute and the Joint Genomes Institute	http://www.phytozome.net/foxtailmillet.php	Zhang et al., [21] Bennetzen et al. [22]

Grape (*Vitis vinifera*)	Consortium	http://www.genoscope.cns.fr/externe/GenomeBrowser/Vitis/	Jaillon et al., 2007 [23]

*Jatropha curcas* L.	Consortium	http://www.kazusa.or.jp/jatropha/	Sato et al., 2011 [24]

Lotus (*Lotus japonicas*)	Consortium	http://www.kazusa.or.jp/lotus/	Sato et al., 2008 [25]

Maize (*Zea mays*)	Consortium	http://www.maizegdb.org/	Schnable et al., 2009 [26]

Model Grass (*Brachypodium distachyon*)	Consortium	http://www.brachypodium.org/	International Brachypodium Initiative, 2010 [27]

Mosses (*Physcomitrella patens*)	JGI	http://genome.jgi-psf.org/Phypa1_1/Phypa1_1.home.html	Rensing et al., 2008 [28]

Mouse ear cress (*Arabidopsis thaliana*,* Arabidopsis lyrata*)	The Arabidopsis Genome Initiative (2000)	http://www.arabidopsis.org/	The Arabidopsis Genome Initiative, 2000 [29]; Cao et al., 2011 [30]; Hu et al., 2011 [31]

Papaya (*Carica papaya*)	Consortium	http://www.plantgdb.org/CpGDB/	Ming et al., 2008 [32]

Peach (*Prunus persica*)	International Peach Genome Initiative	http://www.rosaceae.org/peach/genome	International Peach genome initiative 2013 [33]

Pigeon pea (*Cajanus cajan*)	International Initiative for Pigeonpea Genomics (IIPG)	http://www.icrisat.org/gt-bt/iipg/Home.html	Varshney et al., 2011 [34]

Poplar (*Populus trichocarpa*)	JGI	http://genome.jgi-psf.org/Poptr1_1/Poptr1_1.home.html	Tuskan et al., 2006 [35]

Potato (*Solanum tuberosum*)	Consortium (PGSC)	http://www.potatogenome.net/index.php/Main_Page	The Potato Genome Sequencing Consortium, 2011 [36]

Rape seed (*Brassica napus*)	Consortium (MGBP)	http://www.plantgdb.org/BrGDB/	Wang et al., 2011 [37]

Rice (*Oryza sativa *ssp*. indica and japonica*)	Consortium (IRGSP)	http://rgp.dna.affrc.go.jp/E/IRGSP/index.html	Yu et al., 2002 [38]; Goff et al., 2002 [39]

Sorghum (*Sorghum bicolor*)	JGI	http://www.plantgdb.org/SbGDB/	Paterson et al., 2009 [40]

Soybeans (*Glycine max*)	JGI	http://www.phytozome.net/soybean_er.php	Schmutz et al., 2010 [41]

Strawberry (*Fragaria vesca*)	Consortium	http://www.strawberrygenome.org/	Shulaev et al., 2011 [42]

Tomato (*Solanum lycopersicum*)	Consortium	http://solgenomics.net/organism/Solanum_lycopersicum/genome	The Tomato Genome Consortium 2012 [43]

Watermelon (*Citrullus lanatus*)	International Watermelon Genomics Initiative	http://www.iwgi.org/	Guo et al., 2013 [44]

Mung bean (*Vigna radiata*)	Not available	http://vigra.comparative-legumes.org/	Kang et al., 2014 [45]

**Table 2:** List of databases and their information of application to crop plant research community.

Name of databases	Application
AgBase—a unified resource for functional analysis in agriculture	Search and analyze functional genomics datasets in agricultural species

AutoSNPdb—an annotated single nucleotide polymorphism database for crop plants	Identify SNPs from assembled EST sequences for the crops rice, barley, and *Brassica *

BarleyBase—an expression profiling database for plant genomics	Analyze and visualize plant microarray data

BBGD—an online database for blueberry genomic data.	It stores both EST and microarray data and allows scientists to correlate expression profiles with gene function

BIOGEN BASE—CASSAVA	A web accessible resource for investigating cassava phenomics and genomics information

CastorDB—a comprehensive knowledge base for Ricinus communis.	CastorDB provides a user friendly comprehensive resource on castor with particular emphasis on its genome, transcriptome, and proteome and on protein domains, pathways, protein localization, presence of sumoylation sites, expression data, and protein interacting partners

ChromDB—The Chromatin Database	Locate chromatin-associated proteins, including RNAi-associated proteins, for a broad range of organisms

CR-EST—a resource for crop ESTs	Search for sequence, classification, clustering, and annotation data of crop EST projects

CSRDB—a small RNA integrated database and browser resource for cereals	Search for sequence information on rice, maize, and other cereal crops small RNAs

DEBDOM—Database Exploring Banana Diversity of Manipur	The database DEBDOM provides a sophisticated web base access to the details of the taxonomy, morphological characteristics, utility, and sites of collection of Musa genotypes

DRASTIC—Database Resource for the Analysis of Signal Transduction in Cells	Search for information of plant gene expression in response to pathogens and environmental changes

FLAGdb++—A Database for the Functional Analysis of the Arabidopsis Genome	Search and visualize data for high-throughput functional analysis of *Arabidopsis*, rice, and other plant genomes

GabiPD—a plant integrative “omics” database	Search for comprehensive and extensive information on various plant genomes generated by a German collaborative network of plant genomics research

GCP—The Generation Challenge Programme	An online resource documenting stress-responsive genes comparatively across plant species

GDR—Genome Database for Rosaceae	A central repository of curated and integrated genetics and genomics data of Rosaceae, which includes apple, cherry, peach, pear, raspberry, rose, and strawberry

GeneCAT—gene co-expression analysis toolbox	Novel web tools that combine BLAST and coexpression analyses

GeneSeqer@PlantGDB—gene structure prediction in plant genomes	Predict gene structures of plant genomes

GERMINATE	A generic database for integrating genotypic and phenotypic information for plant genetic resource collections

GGT—Graphical GenoTypes	Software for visualization and analysis of genetic data

GrainGenes—The genome database for small-grain crops	Search for molecular and phenotypic information on wheat, barley, rye, triticale, and oats

Gramene—a resource for comparative grass genomics	Curated resource for genetic, genomic, and comparative genomics data for the major crop species, including rice, maize, wheat, and many other plant (mainly grass) species

MaizeGDB—the Community Database for Maize Genetics and Genomics	Search genetic and genomic information about maize

MANET—The Molecular Ancestry Network	Tracing evolution of protein architecture in metabolic networks

Medicago—A database for personalized data mining of the model legume Medicago truncatula transcriptome	Search for integrated genomic, genetic, and biological information on cool season legume *Medicago truncatula* (Mt)

MetaCrop—a detailed database of crop plant metabolism	A database that summarizes diverse information about metabolic pathways in crop plants and allows automatic export of information for the creation of detailed metabolic models

MetaCrop 2.0—managing and exploring information about crop plant metabolism.	It contains information about seven major crop plants with high agronomical importance and two model plants; MetaCrop is intended to support research aimed at the improvement of crops for both nutrition and industrial use

Narcisse—a mirror view of conserved syntenies	A database dedicated to the study of genome conservation

NIASGBdb—National Institute of Agrobiological Sciences Gene Bank DataBase	Find information about agricultural plant genetics and diseases

P3DB—Plant Protein Phosphorylation Database	Find information about protein phosphorylation in plants

Panzea—a database and resource for molecular and functional diversity in the maize genome	Search for information on relationship between genotype and functional phenotype variations

Pepper EST database—in silico exploitation of EST data to extensively score genes of Capsicum annuum	Comprehensive in silico tool for analyzing the chili pepper (*Capsicum annuum*) transcriptome

PIP—a database of potential intron polymorphism markers	A database of potential intron polymorphism markers in plants

PLACE—plant cis-acting regulatory DNA elements	Search for documented motifs found in plant cis-acting regulatory DNA elements

Plant snoRNA database	Search for comprehensive information on small nucleolar RNAs in plants

PlantCARE—a database of plant cis-acting elements	Search for information on plant cis-acting regulatory elements, transcription sites, enhancers, and repressors

PlantTFDB—Plant Transcription Factor Databases	A comprehensive plant transcription factor database

PlantTribes—a gene and gene family resource for comparative genomics in plants	A plant gene family database based on the inferred proteomes of five sequenced plant species: *Arabidopsis thaliana*, *Carica papaya*, *Medicago truncatula*, *Oryza sativa*, and *Populus trichocarpa *

PLecDom—Plant Lectin Domains server	Find information about plant lectin domains

PlnTFDB—Plant Transcription Factor Database	Find information about transcription factors in plants

PmiRKB—Plant MicroRNA Knowledge Base	Find information about plant microRNAs

PMRD—Plant MicroRNA Database	Find information about microRNA sequences and targets in plants

PODB—the Plant Organelles Database	Search a collection of visualized plant organelles and protocols for plant organelle research

POGs/PlantRBP—a resource for comparative genomics in plants	Search for information on putative orthologous proteins among rice, maize, and *Arabidopsis* with emphasis on RNA-binding proteins

PoMaMo—a comprehensive database for potato genome data	Search for comprehensive genomic information on potato

PREP Suite—Predictive RNA Editor for Plants	Use to predict sites of RNA editing in plants

PRGDB—Plant Resistance Genes DataBase	Find information about genes involved in plant defense mechanisms

pssRNAMiner—a plant short small RNA regulatory cascade analysis server	Identify both the clusters of phased small RNAs as well as the potential phase-initiator

RadishBase—a database for genomics and genetics of radish.	A database containing radish pathways predicted from unigene sequences is also included in RadishBase

RoBuST—an integrated genomics resource for the root and bulb crop families Apiaceae and Alliaceae.	The RoBuST database has been developed to initiate a platform for collecting and organizing genomic information useful for RBV (root and bulb vegetables) researchers

SALAD—Surveyed contained motif ALignment diagram and the Associating Dendrogram	Perform systematic comparison of proteome data among species

SGN—SOL Genomics Network	A comparative map viewer dedicated to the biology of the Solanaceae family

Shanghai RAPESEED Database—a resource for functional genomics studies of seed development and fatty acid metabolism of *Brassica *	Find information on EST, gene expression profiles, and bioresources for the promotion of functional genomics studies and quality breeding of *Brassica* crops

SolRgene—an online database to explore disease resistance genes in tuber-bearing Solanum species	The SolRgene database contains data on resistance to *P. infestans* and presence of R genes and R gene homologues in Solanum section Petota

SoyBase—USDA-ARS soybean genetics and genomics database	Find genetic information about soybeans

SoyTEdb—a comprehensive database of transposable elements in the soybean genome.	SoyTEdb provides resources and information related to transposable elements in the soybean genome, representing the most comprehensive and the largest manually curated transposable element database for any individual plant genome completely sequenced to date

SoyXpress—a database for exploring the soybean transcriptome	A soybean gene expression and transcription database

Sputnik—a database platform for comparative plant genomics	Search for ESTs from over 20 different plant species

TFGD—Tomato Functional Genomics Database	Find information about tomato genes

The Adaptive Evolution Database (TAED)—a phylogeny based tool for comparative genomics	Search for information on adaptive evolution in gene families of higher plants and chordate

The Legume Information System (LIS)—an integrated information resource for comparative legume biology	Search for integrated genetic and molecular data from multiple legume species

The Plant DNA *C*-values Database	Search for information on plant DNA *C*-values and genome sizes

The Plant Ontology Database—a resource for plant structure and developmental stages	View, search, and query plant ontology terms

The PlantsP Functional Genomics Database	Search for information on plant kinases and phosphatases

The TIGR Maize Database	Search for annotated genomic sequences of maize

The TIGR Plant Repeat Databases—A Collective Resource for the Identification of Repetitive Sequences in Plants	Identify, classify, and analyze repetitive sequences in plant genomes

TomatEST database—in silico exploitation of EST data to explore expression patterns in tomato species	Find expressed sequence tag (EST)/cDNA sequence information from different libraries of multiple tomato species

TriMEDB—a database to integrate transcribed markers and facilitate genetic studies of the tribe Triticeae.	The Triticeae mapped expressed sequence tag (EST) database

TropGENE-DB—A Multi-tropical Crop Information System	Search for genetic, molecular, and phenotypic data of tropical crop species

TropGENE-DB—A Multi-tropical Crop Information System	Search for genetic, molecular, and phenotypic data of tropical crop species

UK CropNet—a collection of databases and bioinformatics resources for crop plant genomics	Search sequences and genomic information on crop plants

WhETS—Wheat Estimated Transcript Server	A tool to provide the best estimate of hexaploid wheat transcript sequence
